# High Electrochemical Performance of Nanotube Structured ZnS as Anode Material for Lithium–Ion Batteries

**DOI:** 10.3390/ma11091537

**Published:** 2018-08-26

**Authors:** Wen Zhang, Junfan Zhang, Yan Zhao, Taizhe Tan, Tai Yang

**Affiliations:** 1 School of Materials Science and Engineering, Research Institute for Energy Equipment Materials, Hebei University of Technology, Tianjin 300130, China; zhangwen@hebut.edu.cn (W.Z.); 18722593259@163.com (J.Z.); 2 Synergy Innovation Institute of GDUT, Heyuan 517000, Guangdong, China; tztansii18@163.com

**Keywords:** lithium-ion batteries, zinc sulfide, nanotubes, anode material, electrochemical performance

## Abstract

By using ZnO nanorods as an ideal sacrificial template, one-dimensional (1-D) ZnS nanotubes with a mean diameter of 10 nm were successfully synthesized by hydrothermal method. The phase composition and microstructure of the ZnS nanotubes were characterized by using XRD (X-ray diffraction), SEM (scanning electron micrograph), and TEM (transmission electronic microscopy) analysis. X-ray photoelectron spectroscopy (XPS) and nitrogen sorption isotherms measurements were also used to study the information on the surface chemical compositions and specific surface area of the sample. The prepared ZnS nanotubes were used as anode materials in lithium-ion batteries. Results show that the ZnS nanotubes deliver an impressive prime discharge capacity as high as 950 mAh/g. The ZnS nanotubes also exhibit an enhanced cyclic performance. Even after 100 charge/discharge cycles, the discharge capacity could still remain at 450 mAh/g. Moreover, cyclic voltammetry (CV) and electrochemical impedance spectroscopy (EIS) measurements were also carried out to evaluate the ZnS electrodes.

## 1. Introduction

In recent decades, lithium-ion batteries play an increasingly dominating role in portable electronic devices due to the fact that they have the advantages of long service life, high energy density, high reversible capacity, and environmental friendliness [1]. Graphitic materials as a conventional anode material in lithium-ion batteries are extensively used for their good electrochemical properties and their structural stability during charge–discharge cycling [2]. However, traditional graphitic carbon materials severely hinder the development of lithium-ion batteries due to their low theoretical capacity (372 mAh/g) [3,4]. In order to meet energy storage needs, it is necessary to exploit new types of anode materials to replace carbon materials. Metal sulfides—such as CuS, MoS_2_, NiS, and ZnS—also have been used as anodic electrode materials in lithium-ion batteries [5,6,7,8]. For example, CuS/graphene composite have a good charge–discharge cycling performance; however, its initial discharge capacity was only 627 mAh/g [9]. NiS-carbon nanofiber films have worse electrochemical properties, and its discharge capacity decayed below 100 mAh/g after 40 cycles [10]. MoS_2_ nanowall/graphene has a stable discharge capacity of about 700 mAh/g [11], which is still unimpressive. ZnS, by contrast, is viewed as a very promising alternative to carbon anode material due to its high theoretical capacity (962.3 mAh/g) [12]. Unfortunately, some drawbacks hinder its commercialization process. The main problem is significant volume changes during its charging and discharging processes, which leads to a great capacity fade upon cycling [13]. Moreover, normal ZnS particles have poor electrical conductivity, as a result, anode electrodes made using unmodified ZnS suffer from a poor cyclic and rate performance [14].

There are two possible ways to solve above mentioned problems. For one thing, we should focus on the synthesis of nano-sized particles, which can effectively adapt to volume changes during the charge–discharge progress [15,16,17]. For another, it is an effective method to combine nano-structured ZnS particles with conductive carbon coating to increase conductivity of anodic materials in lithium-ion batteries [18]. He et al. [14] prepared ZnS/C composites by a combined precipitation with carbon coating method and applied them as anode material for lithium-ion batteries. Du et al. [12] also synthesized nanocrystalline ZnS/C with core/shell structure by using a simple solvothermal process and an annealing process. These studies have made some progress in development of anodic materials for lithium-ion batteries. Nevertheless, the above-mentioned preparation methods of nanocrystalline ZnS/C composites are complicated and costly. 1-D ZnS nanotubes also have been proved to be a promising candidate material [19,20]. It is well-known that active materials in anodes with large surface areas can increase the contact area between electrolyte and electrode materials, thereby enhancing energy storage density [21]. Moreover, nano-materials can also shorten the transport path of conductive ions, and the electrodes will not be destroyed even though a large volume change of ZnS occurs in the charge–discharge process [22,23]. It was reported that nanotubes can expand radially as well as longitudinally to mitigate the stress, which would make them more suitable for high rate applications [24].

In order to further investigate the electrochemical performance of nano-structured sulfides, ZnS nanotubes were prepared by hydrothermal method by using ZnO nanorod arrays as sacrificial template. The prepared ZnS nanotubes exhibit a well rate discharge performance. The discharge capacity of ZnS nanotubes is as high as 950 mAh/g in the first cycle, and it still remains at 450 mAh/g after 100 charge/discharge cycles.

## 2. Materials and Methods

### 2.1. Synthesis of ZnS Samples

Firstly, 25 mmol of Zn(NO_3_)_2_∙6H_2_O and 50 mL of polyvinyl pyrrolidone (PVP) aqueous solution (0.1 wt %) were mixed with a certain amount of deionized water to get Zn(NO_3_)_2_ solution of 0.05 mol/L. At the same time, hexamethylenetetramine (C_6_H_12_N_4_) solution (50 mL, 0.1 wt %) was also prepared. Above two solutions were mixed, heated, and stirred in a beaker-flask at 90 °C for 16 h. Then the white products were collected and washed by deionized water and ethanol three times, and the precipitate was dried in a vacuum oven at 70 °C for 12 h. Finally, pure ZnO nanorods were obtained.

Subsequently, the ZnS nanotubes were synthesized by hydrothermal method by using the ZnO nanorods as template. The prepared pure ZnO nanorods were dispersed in 20 mL of ethylene glycol (C_2_H_6_O_2_) solution, stirring and sonicating for 20 min. After that, thioacetamide (CH_3_CSNH_2_) was dripped into the above-mentioned ZnO suspension. The mixture solution was transferred to a Teflon-lined stainless-steel autoclave and placed into an oven maintained at 145 °C for 10 h. After this reaction, the ZnO/ZnS nano composites were collected and washed three times using deionized water and pure ethanol. Then, 2 g of ZnO/ZnS nano composites were added into 50 mL of 10 M NaOH aqueous solution and stirred for 2 h at room temperature to remove ZnO cores. The products were collected and dried at 80 °C for 10 h, and then white ZnS nanotubes were obtained.

### 2.2. Sample Characterizations

XRD method was used to analyze the phase composition and crystal structure of the sample. The tests were performed at a scanning rate of 2°/min in the 2*θ* range from 20° to 90° by using an X-ray diffractometer (SmartLab Rigaku Corporation, Tokyo, Japan). Identification of the species was computer aided. The microstructure and corresponding selected area electron diffraction (SAED) patterns for the ZnS nanotubes were also performed by using SEM (Hitachi S-4800) and TEM (JEOL-2010). Nitrogen sorption isotherms and Brunauer–Emmett–Teller (BET) surface area were measured at 423 K with a V-Sorb 2800P analyzer (GAPP, Beijing, China). XPS (Thermo Fisher Scientific, Waltham, MA, USA) measurements were conducted to evaluate the chemical states of elements in the sample. 

### 2.3. Electrochemical Measurements

The electrochemical behaviors of the ZnS nanotubes were characterized by using CR 2025 coin cell. In order to prepare working electrodes (anodic electrodes), a slurry was mixed by using 70 wt % of ZnS nanotube powder, 15 wt % of carbon black and 15 wt % of polyvinylidene fluoride. The mixture was grinded for 40 min and dissolved in N-methyl-2-pyrrolidone (NMP), and the obtained slurry with a thickness of 0.1 mm was blade cast onto Cu foil. Then the prepared electrode material was dried at 70 °C for 12 h. After that, the dried electrodes were punch into coins in an argon-filled (99.999%) glove box. The ZnS loading amount of each electrode sheet was approximately 2.5 mg/cm^2^. Pure lithium metal foils were used as reference anode, and microporous polypropylene as a separator. The electrolyte was a solution of 1 mol/L LiPF_6_ in ethylene carbonate (C_3_H_4_O_3_) and dimethyl carbonate (C_3_H_6_O_3_) with a volume ratio of 1:1. The charging and discharging measurements and cycle life tests of the prepared coin cells were carried out by using a multichannel battery testing system (Neware BTS4000). Considering the theoretical capacity (962.3 mAh/g) of ZnS [12], the charge–discharge current density of 962.3 mA/g was defied as 1 C. After the 100th charge–discharge cycle, the cells were dismantled to collect the anode materials. Then the anode materials were soaked in N-methyl-2-pyrrolidone (NMP) for 4 h to remove the binder and conductive agent. The phase structure and micro morphology of the collected ZnS nanotubes were also carried out by XRD and SEM. The charge–discharge voltage ranged from 0.05 V to 3.00 V. CV curves for the first three cycles were performed by an electrochemical workstation (Princeton, Versa STAT 4) at a scan rate of 0.1 mV/s in a voltage range of 0.01–3.00 V. The EIS measurements were also performed by the same electrochemical workstation with a frequency range of 10 kHz–10 mHz with a small sinusoidal perturbation of 10 mV. 

## 3. Results and Discussion

### 3.1. Structural and Composition Characterization

**Figure 1:**
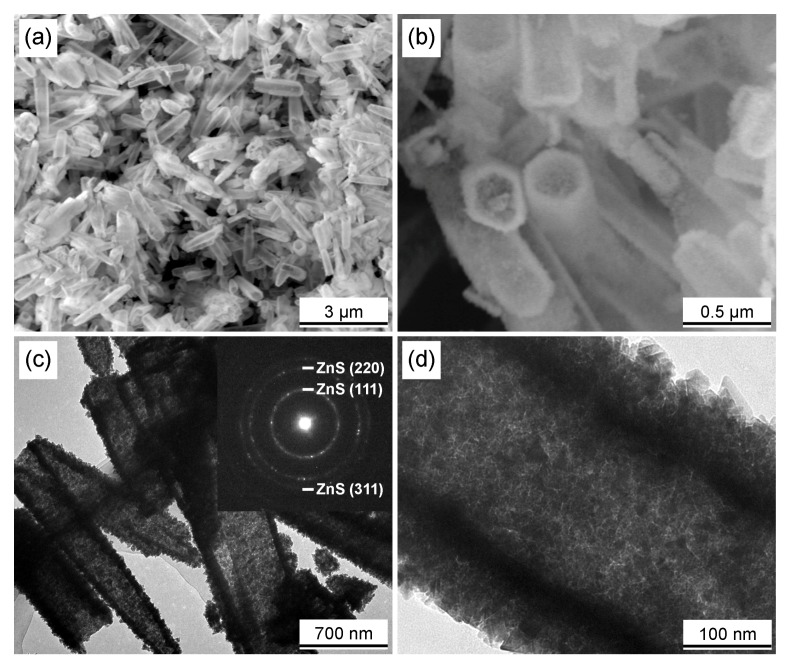
(**a**,**b**) SEM images of the prepared ZnS nanotubes; (**c**,**d**) TEM images together with corresponding SAED patterns of the ZnS nanotubes.

SEM and TEM analysis were used to clarify the fine microstructures and morphologies of the ZnS sample. Figure 1a,b shows the SEM images of the ZnS nanotubes. It can be easily observed that the morphology of the ZnS sample is a kind of hollow micro tube, with the tube wall thickness of about 80 nm and the length was 1–2 μm. Detailed structural information of the ZnS sample was further investigated by TEM, results are shown in Figure 1c,d. Clearly, the nanotubes have a rough surface. The SAED pattern confirms the existence of ZnS. The three bright ED patterns correspond to the (111), (220), and (311) lattice plane of ZnS. Moreover, it can be observed from the high-resolution image shown in Figure 1d that the nanotubes are mainly composed of nanocrystals. This type of nanostructure contributes to the enhancement of electrochemical performance for the electrodes.

**Figure 2:**
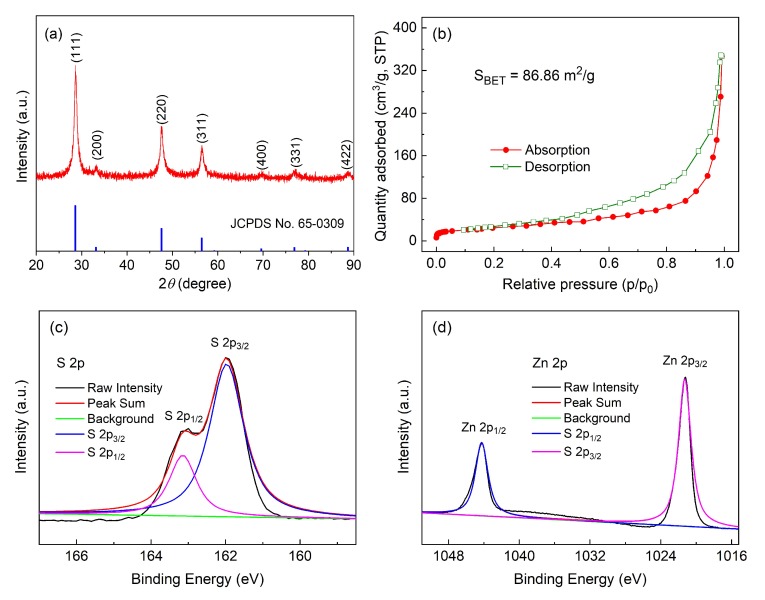
(**a**) XRD pattern of the ZnS nanotubes; (**b**) N_2_ adsorption-desorption isotherm of the ZnS nanotubes; (**c**,**d**) XPS analysis for the ZnS nanotubes.

The XRD pattern of the synthesized ZnS nanotube sample and corresponding JCPDS data are shown in Figure 2a. Sharp diffraction peaks indicate good crystallinity of the sample. All of the diffraction peaks correspond well with the data of ZnS (JCPDS no. 65-0309). The three major diffraction peaks located at 2*θ* = 28.5°, 47.5°, and 56.3° correspond to (111), (220), and (311) crystal planes of ZnS. In order to know actual surface area of the ZnS nanotubes, N_2_ adsorption and desorption isotherms are carried out, results are shown in Figure 2a. Type IV isotherm curve is observed with hysteresis loop at higher pressure, indicating a large number of meso-pores present in the sample [25]. The BET specific surface area of the sample was as high as 86.86 m^2^/g. The XPS measurement was also conducted to obtain the information on the surface chemical compositions and the valence states of corresponding elements in the sample. From Figure 2c, it can be seen that the XPS spectra of S 2p was divided into two peaks centered at 163.1 and 162.0 eV, corresponding to S 2p_1/2_ and S 2p_3/2_ states [26]. Figure 2d depicted the XPS spectrum of the Zn 2p peaks centered at 1044.2 and 1021.3 eV, which associated with Cu 2p_1/2_ and Cu 2p_3/2_, respectively [27].

### 3.2. Electrochemical Performance

**Figure 3:**
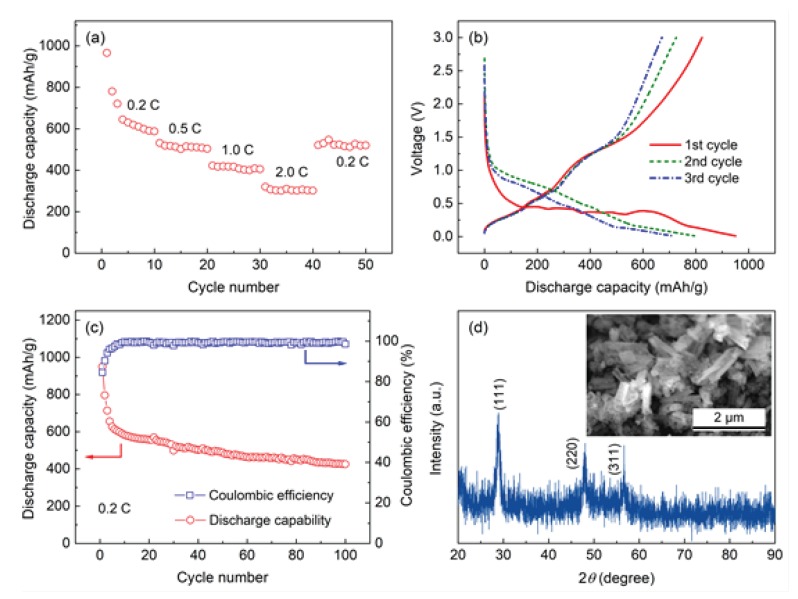
(a) Rate discharge capability of the ZnS electrodes together with the SEM images of ZnS nanotubes; (b) the first three galvanostatic charge/discharge profiles of the ZnS electrodes; (c) cycling performance and coulombic efficiency of the ZnS electrodes; (d) XRD pattern and SEM images of the ZnS nanotubes after 100th charge–discharge cycles.

The discharge rate capabilities of the ZnS electrodes were tested at different current densities, as shown in Figure 3a. It can be observed that the discharge capacity of the ZnS electrode was steadily and has the high reversible capacities of 610, 500, 410, and 320 mAh/g at the discharge current density of 0.2, 0.5, 1, and 2 C. The specific capacity of the electrode is nearly recovered to its initial value in the case of the current density is goes back abruptly from 2 C to 0.2 C, further proving good reversibility and excellent cycling stability of the ZnS nanotube electrodes. The good rate discharge performance of the ZnS nanotubes is mainly due to the shortened lithium-ion diffusion distance and enhanced structural stability of the unique nanotubular structure [28]. Moreover, the hollow tubular structure can greatly increase the surface area of the material, which increases contact area between electrolyte and ZnS electrode material [29].

The charge–discharge profiles of the ZnS electrodes were evaluated by using galvanostatic method at the current density of 0.2 C, as shown in Figure 3b. During the initial discharge process, a typical slope can be clearly seen when the voltage was higher than 0.5 V. A major discharge voltage plateau can be observed at about 0.45 V, which represents to the lithiation reaction of ZnS nanotubes [30]. In the next few cycles, several typical charge/discharge stages can be observed, which could be due to the electrochemical activation of the system and tend to be stable [31]. The capacity fade during the first cycle can be result from the decomposition of electrolyte, which leads to the redistribution of the active materials [32]. In the following charge–discharge process, the ZnS electrode exhibits the same curves, indicating that the ZnS electrode has become stable. According to He et al. [14], the lithium insertion/extraction mechanism of ZnS active electrode material can be expressed: (*x* + 2)Li^+^ + ZnS + (*x* + 2)e^−^ ↔ Li_2_S + Li*_x_*Zn.

Figure 3c presents the cycling performance of the ZnS electrodes. It can be observed that it has an initial reversible discharge capacity of 950 mAh/g and its capacity keeps steady even after 100 cycles. In order to evaluate the charge–discharge efficiency, the coulombic efficiency of the electrodes was also exhibited in Figure 3c. The coulombic efficiency is defined as the percentage of discharge and charge capacity in one cycle. It is seen from Figure 3c that the coulombic efficiency is almost 100% after about 10 cycles. Therefore, it can be concluded that the ZnS nanotube electrodes have a good cycling stability, meanwhile delivering a quite high reversible capacity. This is due to the fact that the ZnS nanotubes possess a large specific area, which provides more active sites for the lithium ions [33]. That is, during the charge or discharge processes, tube shaped ZnS can accommodate more lithium ions [33]. Moreover, 1-D ZnS nanotubes can shorten the lithium ion diffusion distance [34]. At the same time, nanotube structure has a beneficial effect on the volume expansion/shrinkage of ZnS during charge–discharge process [35]. The above reasons are why the ZnS nanotube electrodes have a good rate discharge capability and long cycle life. 

In order to further investigate the structural changes of the ZnS nanotubes during charge– discharging process, the phase composition and microstructure for the ZnS nanotubes after 100 charge–discharge cycles were performed by using XRD and SEM. As shown in Figure 3d, three diffraction peaks can be clearly observed, which corresponds to the (111), (220), and (311) crystal. However, the intensity of the diffraction peaks is smaller than that of the prepared ZnS nanotubes before electrochemical cycle (Figure 2a). This may be due to the lower testing sample quantity. In addition, the SEM image of the sample after 100 charge–discharge cycles shows that the ZnS nanotubes still maintain a tube shape structure, together with some small amount of broken tube walls. Clearly, the ZnS nanotubes have a good structural stability during charge–discharge cycling, which leads to a good discharge capacity retention ratio.

**Figure 4:**
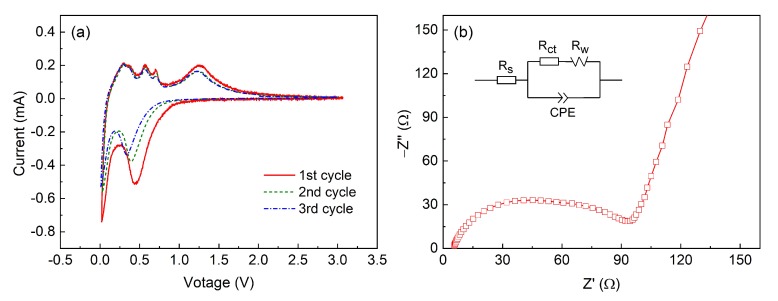
(**a**) CV curves of the ZnS nanotube electrode at a scan rate of 0.1 mV/s; (**b**) Nyquist plots of the ZnS electrodes.

Figure 4a shows the CV curves of the ZnS nanotube electrode at a scan rate of 0.1 mV/s between 0.01 to 3 V for the first, second, and third discharge/charge processes. In the first discharge process, two broad reduction peaks can be observed in the potential range of 1.1–0.25 V and 0.25–0.05 V, respectively. The first reduction peak ranging from 1.1–0.25 V can be ascribed to the decomposition of ZnS into Zn and the formation of Li_2_S, while the second reduction peak ranging from 0.25–0.05 V corresponds to the subsequent reaction of lithium ions with Zn metal [14]. Decomposition of the electrolyte and formation of the solid electrolyte interface on the surface of electrode particles also occurs during the first cathodic cycle, which causes part of the irreversible capacity [12,14]. During the anodic scanning process, anodic peaks located at 0.28, 0.37, 0.57, and 0.71 V are observed, which is attributed to the multi-step de-alloying process of Li-Zn (LiZn, Li_2_Zn_3_, LiZn_2_, and Li_2_Zn_5_) [12]. Another bigger anodic peak at 1.26 V is attributed to the back-conversion of Zn and Li_2_S into ZnS [12,14]. In the following cycles, the CV curves do not change position or intensity of any of the peaks, indicating that the ZnS nanotubes have a good reversibility.

To further confirm the electrochemical performance, the EIS studies of the ZnS nanotube electrodes were conducted, results are shown in Figure 4b. A semicircle and a slope line can be observed for both of the samples. As reported by Cheng et al. [36], the semicircles relate to the charge transfer resistance which resulted from charge transfer through the electrode/electrolyte interface, while the slope line corresponds to Warburg impedance over Li^+^ diffusion in the solid materials. The impedance data were analyzed by fitting the curves with equivalent electrical circuit shown in the inset of Figure 4b, where R_s_ and R_ct_ indicate electrolyte resistance and charge transfer resistance, respectively; CPE stands for the corresponding constant phase elements; and R_W_ represents the diffusion-controlled Warburg impedance. The charge-transfer resistance of the as-synthesized ZnS nanotube electrodes is 88 Ω, which is much lower than the pure ZnS nanoparticles (199.5 Ω) reported by Zhang et al. [37]. Moreover, the ZnS nanotube electrode has a higher slope, which means the Li^+^ diffusion in the ZnS nanotubes is much faster than that of ZnS nanoparticles. Clearly, ZnS nanotube electrodes exhibit a good electrochemical performance.

## 4. Conclusions

Comprehensive analysis—using XRD, SEM, TEM, and XPS—indicates that the ZnS nanotubes were successfully prepared via a hydrothermal method. The BET specific surface area of the sample is as high as 86.86 m^2^/g. The ZnS nanotube electrodes exhibit a good lithium ion storage capability. The first discharge capacity was about 950 mAh/g, and it could still remain at 450 mAh/g after 100 charge/discharge cycles. This is due to the fact that the ZnS nanotubes have a good structural stability during charge–discharge cycling. In a word, this work provides a rational strategy to advance the ZnS anode electrochemical performance in lithium-ion batteries applications.
